# The absence or the pharmacological blockade of the aryl hydrocarbon receptor promotes neuroprotection in the hippocampus after an ischemic insult

**DOI:** 10.1371/journal.pone.0338936

**Published:** 2025-12-26

**Authors:** Rolando Castañeda-Arellano, Lucia García-Lara, Quetzalli D. Angeles-López, Francisca Pérez-Severiano, Guillermo Elizondo-Azuela, Sergio H. Dueñas-Jimenez, Sirenia González-Pozos, Irene G. Aguilar-García, Jose Segovia

**Affiliations:** 1 Laboratorio de Farmacología, Centro de Investigación Multidisciplinario en Salud, Centro Universitario de Tonalá, Universidad de Guadalajara, Tonalá, México; 2 Departamento de Fisiología, Biofísica y Neurociencias, Centro de Investigación y de Estudios Avanzados del IPN, Ciudad de México, México; 3 Laboratorio de Neurofarmacología Molecular y Nanotecnología, Instituto Nacional de Neurología y Neurocirugía Manuel Velasco Suárez, Ciudad de México, México; 4 Departamento de Biología Celular, Centro de Investigación y Estudios Avanzados del IPN, México; 5 Departamento de Neurociencias, Centro Universitario de Ciencias de la Salud, Universidad de Guadalajara, Guadalajara, México; 6 Laboratorio Nacional de Servicios Experimentales, Microscopía Electrónica, Centro de Investigación y Estudios Avanzados del IPN, Ciudad de México, México; The Islamic University, IRAQ

## Abstract

The aryl hydrocarbon receptor (AhR) is a transcription factor that acts as a nuclear receptor mediating xenobiotic metabolism and environmental responses. Recent evidence suggests that the AhR is implicated in maintaining homeostasis or triggering pathological effects by modulating different physiological responses in the nervous system. However, the underlying mechanisms of the AhR-induced effects in the hippocampus remain unclear. Herein, we report a previously unknown role for the AhR in the pathogenesis of a transient global ischemic insult. Comparative analysis of the expression of HIF-1α, nNOS, iNOS, IL6, KAT II, and claudin 5 in the hippocampus of AhR^+/+^ and AhR^-/-^ mice after bilateral common carotid artery occlusion revealed differential modulation. We report that AhR^-/-^ mice significantly reduced the blood-brain barrier (BBB) dysfunction and neuronal death in hippocampus-CA1 caused by the ischemic insult, inducing neuroprotection. In addition, blocking the AhR with Resveratrol (a competitive antagonist) decreased neuronal degeneration and preserved the integrity of the BBB in the hippocampus associated with a neuroprotective effect. In summary, we showed that the AhR is an essential regulator for maintaining the BBB in the hippocampus and responds to inflammatory conditions and oxidative stress; further pharmacological targeting of AhR signaling may reduce the pathologic changes caused by an ischemic insult in the hippocampus.

## Introduction

The aryl hydrocarbon receptor (AhR) is ubiquitous, with a broad-spectrum homeostatic role; it contributes to both physiological and pathological phenomena by modulating signaling pathways leading to regulate cell cycle, apoptosis, and cell differentiation [1,2]. The AhR belongs to the family of transcription factors with a basic helix loop helix-Per-Arnt-Sim domain (bHLH/PAS). It is a crucial regulator of several xenobiotic-metabolizing enzymes (XME), and it is activated by exogenous ligands such as 2, 3, 7, 8-tetrachlorodibenzo-p-dioxin (TCDD) and benzo (a) pyrene or by endogenous ligands such as kynurenine and kynurenic acid (KYNA) [3,4]. In the central nervous system (CNS), the AhR is expressed in several regions, including the cerebellum, hippocampus, and cortex [5]. Specifically, the AhR is expressed in neurons, astrocytes, and vascular endothelial cells. Moreover, the AhR-knockout induces a neuroprotective effect after a neurotoxic injury, and in a transgenic model of Huntington´s disease [6,7], and it has also been related to pathophysiological phenomena such as ischemia, trauma, and neurodegenerative diseases [8]. However, the molecular mechanisms underlying the functions of the AhR are not entirely understood yet.

Disorders characterized by an ischemic event such as myocardial infarction, stroke, and peripheral vascular disease, are a leading cause of morbidity and mortality, with a significant health burden worldwide [9]. The mechanisms of cerebral ischemic injury include events such as excitotoxicity, oxidative stress, and inflammation, which are the main routes that regulate selective signaling systems leading to neuronal cell death and increased permeability of the Blood-Brain Barrier (BBB) [10]. Interestingly, the response to low oxygen availability is mediated by the hypoxia-inducible factor (HIF-1α) that promotes gene expression associated with cell survival [11]. The AhR and HIF-1α are necessary sensors of the response to the environment since they share the same interactions with other proteins like ARNT; thus, interference between these transcription factors may occur.

On another note, Resveratrol (Rsv) promotes a variety of beneficial effects such as anti-inflammatory, antioxidant, and regulator apoptosis [12]. In the CNS, Rsv reduces neurological damage in models of neurodegenerative diseases and brain injury [13,14]. The Rsv treatment diminishes hypoxia-ischemia induced childhood hippocampal dysfunction reduces brain edema and some status epilepticus effects on the hippocampus [15,16]; it improves dysfunctional BBB permeability activating SIRT1 in a subarachnoid hemorrhage model [17] and reduces the Amyloid beta-mediated neurotoxicity trough the AMPK signaling in human neural stem cell (hNSCs) [18].

Interestingly, Rsv is a competitive antagonist of the AhR, preventing gene transactivation [19] and allowing changes in signaling-mediated pathways implied in neuroprotection. Multiple studies using biochemical, reporter, and gene-expression assays have demonstrated that Rsv behaves as a competitive antagonist of the AhR [20–22]. It promotes AhR nuclear translocation and DNA binding at dioxin-responsive elements but prevents the subsequent transactivation of canonical AhR target genes as CYP1A1 and CYP1B1, thereby inhibiting ligand-induced expression [23]. However, Rsv is a pleiotropic small molecule with multiple documented molecular targets and cellular effects beyond AhR antagonism, including modulation of SIRT1, AMPK, COX, and redox pathways; these off-target activities complicate a straightforward interpretation that observed protective effects are exclusively AhR-mediated [24,25]. In the present work, we studied the hippocampus region of mice knock-out for the AhR after Bilateral Common Carotid Artery Occlusion and treated with Rsv to demonstrate that elimination of the AhR or its blockade by Rsv promotes a neuroprotective effect.

## Materials and methods

### Animals and drug administration

**Fig 1:**
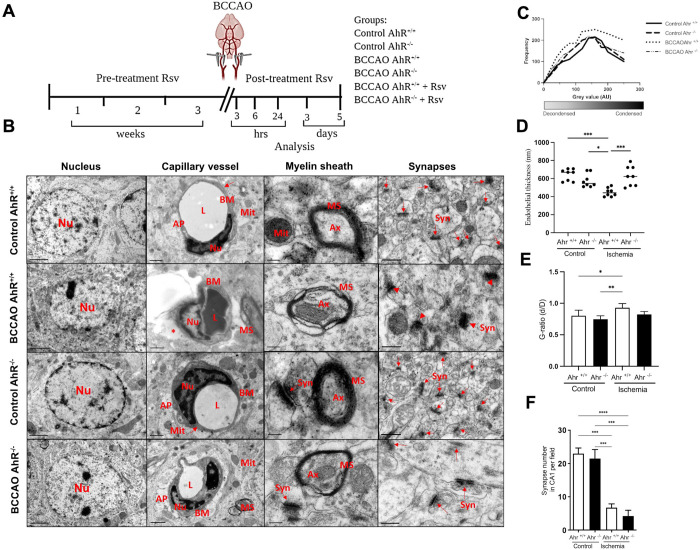
A) Schematic diagram of the BCCAO, treatment with Rsv before the injury. Rsv pre-treatment three weeks before the ischemic event and Rsv post-treatment was performed by day five after ischemia. The analyses were performed at 3, 6, and 24 h and on day five after the injury. **B)** Ultrastructural changes at the nucleus, capillary vessel, myelin sheath, and synapses in the hippocampus CA1 after BCCAO. **C)** Quantification of chromatin condensation levels of dark and light pixels from images inside the nucleus, representing condensed and decondensed chromatin, of electron microscopy images **D)** Multiple measurements along the endothelial thickness plotted also enabled calculation as a function of lumen area. **E)** Quantification of g-ratio, the ratio of the axon’s diameter to the total diameter of the axon plus its myelin sheath of myelinated axons. **F)** Number of synapses in TEM micrographs. The graphs show significantly changes in the ischemic groups (AhR + /+ and AhR-/-). Data are mean ± SEM, n = 5 per group. *P < 0.05. Mann Whitney test. Nucleus (Nu), scale bar = 2 µm. Vessel; Nucleus (Nu), Mitochondrion (Mit), Basement Membrane (B.M.), Capillary Lumen **(L)**, Astrocyte Process (A.P.), Collapsed A.P. (*). Scale bar = 0.5 µm.

7-week-old male *AhR-null* mice (AhR^-/-^) were generously provided by Dr. Frank J. Gonzalez (National Cancer Institute, Bethesda, MD). The mice were genotyped by PCR, as previously described by Rodriguez-Sosa et al. [26]. Homozygous wild-type mice (AhR^+/+^) from the C57BL/6N genetic background were employed. Mice had access to chow and water *ad libitum* and were placed on a 12 h: 12 h light/dark cycle. Homozygous wild-type (AhR^+/+^) and AhR-null mice (AhR^-/-^) were divided into three different groups: 1) intact groups (control AhR^+/+^ and control AhR^-/-^), 2) Ischemic groups by Bilateral Common Carotid Artery Occlusion (BCCAO), (BCCAO AhR^+/+^ and BCCAO AhR^-/-^) and 3) Ischemic + Resveratrol groups (BCCAO AhR^+/+^ Rsv and BCCAO AhR^-/-^ Rsv). Resveratrol was administered as a pre-treatment 25 mg/kg by oral gavage for three weeks before BCCAO, until five days after injury model as a post-treatment (Fig 1).

The histopathological changes in the hippocampus CA1, from the first- and fifth days post-damage, were analyzed: ultrastructural nuclear changes, alterations in capillary vessels, the effect in the myelinated fiber structures, electron micrographs showing the ultrastructural features of synapses, Fluoro-Jade B staining (FJB) and astrogliosis analysis were also performed. At 3, 6, 24 hours (h), 3, and 5 days post-BCCAO, the expression of HIF-1α, nNOS, and iNOS in both AhR^+/+^ and AhR^-/-^ mice by Western blot and immunoblotting of nNOS and iNOS KATII, Claudin 5 and IL-6 were analyzed. Activity of mice in the open field test after BCCAO was evaluated at 1 and 5 days (Fig 1).

All animal procedures were conducted in accordance with the Guide for the Care and Use of Laboratory Animals, as established by the U.S. National Institutes of Health, the Mexican Regulation of Animal Care and Maintenance (NOM-062-ZOO-1999, 2001), and the Internal Committee for the Use of Laboratory Animals of CINVESTAV. For tissue collection, mice were deeply anesthetized with an intraperitoneal injection of sodium pentobarbital at 90 mg/kg. Once a deep plane of anesthesia was confirmed—indicated by the absence of both pedal and corneal reflexes—transcardial perfusion was performed using 0.9% saline, followed by 4% paraformaldehyde in 0.1 M phosphate buffer (pH 7.4) for histology and immunohistochemistry. For western blot analysis, mice were euthanized by decapitation while under deep anesthesia. All efforts were made to minimize suffering during these procedures.

### Bilateral Common Carotid Artery Occlusion Model (BCCAO)

Mice were anesthetized with a mixture of ketamine (90 mg/kg) and xylazine (10 mg/kg), and body temperature was monitored and maintained in a range between 37–37.5 °C using a thermal mattress. The shaved area was sterilized with an iodine solution, and immediately an incision in the middle line of the neck was performed, and both common carotid arteries were dissected. The occlusion of the two common cerebral arteries was performed as previously described [27,28], using clamps in each carotid for 15 min, subsequently removing the clamps and restoring blood circulation to the brain (event of reperfusion). For evaluating protein expression after injury, mice were analyzed at 3, 6, 24 h, and 3-, and 5 days post-injury, and histopathological data were observed at the first- and fifth days post-injury (Fig 1).

### Transmission electron microscopy (TEM)

We obtained biopsy samples from the CA1 area of the hippocampus for transmission electron microscopy analysis. On the fifth day after reperfusion, mice were deeply anesthetized and perfused with a mixture of 4% paraformaldehyde and 2.5% glutaraldehyde in Phosphate-buffered saline (PBS). Fifty µm thick coronal sections were cut on a vibratome (Thermo Scientific, HM650V) and returned to be fixed again with glutaraldehyde. The samples were then post-fixed in 1% osmium tetroxide (Merck-Sigma, O5500) for two h at 4°C. Samples were gradually dehydrated in ethyl alcohol (50, 60, 70, 80, 90, 100% per 10 min each) and embedded in Spurr’s resin (Merck-Sigma, EM0300). The polymerization was performed in an oven at 60^o^C for 48 hours. Ultra-sections were made with an ultramicrotome (Leica, UC6, Germany) and placed on 200-mesh copper grids. Thin sections were stained with uranyl acetate for two hours and with lead citrate (Merck-Sigma, 15326) for 10 min. Thin sections were analyzed using a transmission electron microscope JEM 1400EX (JEOL, Japan). For a detailed description of electron microscopy analysis see S1 File.

### Fluoro-Jade B staining

Fluoro-Jade B stain was used to visualize degenerating neurons in the hippocampal CA1 region. Briefly, mice were deeply anesthetized with pentobarbital, the first group on the first day and the second group on the fifth day after reperfusion. Then, mice were transcardially perfused with 0.9% saline solution and 4% paraformaldehyde in 0.1 M phosphate buffer (pH 7.4). Brains were removed and placed in the same ﬁxative solution at 4°C. Coronal sections 30 µm thick were cut on a vibratome (Thermo Scientific, HM650V). Slides were heated at 50°C for 10 min before staining, and immersed in 70% ethanol for 2 min, followed by 2 min in distilled water. After incubation in a 0.06% solution of potassium permanganate for 15 min, slides were rinsed twice in distilled water for 2 min. Then they were transferred to a solution containing 0.0004% Fluoro-Jade B (Merck Millipore, AG310) and 0.1% acetic acid for 30 min, rinsed for 2 min in distilled water, dried for one h in the dark room, immersed in xylene for 10 min and then coverslipped. Examination of Fluoro-Jade B was performed using fluorescence microscopy (Olympus BX51).

### Immunofluorescence

We evaluated the astrogliosis caused by the BCCAO using GFAP-immunofluorescence on the fifth day after reperfusion. Tissues were obtained as described above. Free-floating 30 μm, sections were incubated at room temperature for 30 min in PBS 1x/Triton X-100 0.2%. Next, sections were incubated for 1 hr in PBS 1x/BSA 1%, and then with a GFAP antibody (1: 750, DAKO, Z0334, RRID: AB_10013382) overnight and subsequently incubated with the secondary antibody (FITC anti-rabbit IgG) for two h. Sections were analyzed with a laser confocal scanning microscope (Leica TCS-SPE) with a 40-oil immersion objective. The Leica LAS AF lite software (Leica Microsystems, Wetzlar, Germany) was used for the image analysis.

### Western blot analysis

Total protein was obtained from the hippocampus CA1 region at 3, 6, 24 h, 3, and 5 days post-BCCAO using a lysis buffer containing a protease inhibitor cocktail (Complete, Roche Diagnostics, Darmstadt, Germany), and protein quantification was performed using the bicinchoninic acid method (Pierce, Rockford, IL). To determine protein expression in specific subcellular compartments, nuclear and cytoplasmic fractions were isolated from hippocampal tissue using a fractionation method of hypotonic buffer solution (20 mM Tris-HCl (pH 7.4), 10 mM NaCl, 3 mM MgCl_2_). The purity of fractions was verified by probing for the nuclear marker Lamin a/c and the cytoplasmic marker actin (See Supplementary Methods in S1 File). Aliquots containing 50 μg of protein were separated on 12% SDS-PAGE gels and transferred into polyvinylidene difluoride (PVDF) membranes (BioRad, Hercules, CA). Membranes were blocked with 5% nonfat milk/TBST (0.05% Tween-20, TBS) for 1 hour at room temperature and incubated with the primary antibodies overnight at 4°C. Blots were then incubated with a peroxidase-coupled secondary antibody followed by enhanced chemiluminescence detection (Perkin Elmer), according to the manufacturer’s instructions. The primary antibodies were anti-HIF-1α (1:100 Santa Cruz Biotechnology sc-13515, RRID: AB_627723), anti-nNOS (1:250 Santa Cruz Biotechnology, sc-5302, RRID: AB_626757), anti-iNOS (1:250 Santa Cruz Biotechnology, sc-649, RRID: AB_631833), anti-KAT II (1:200, Santa Cruz Biotechnology, sc-67376, RRID: AB_2219610), anti-claudin 5 (1:250 Abcam, ab15106, RRID: AB_301652), anti-IL-6 (1:100 Santa Cruz Biotechnology, sc-1265, RRID: AB_2127470) anti-β actin (1:5000, donated by Garcia-Tovar [29] and anti-Lamin A/C (Santa Cruz Biotechnology, sc-7292). Images from ﬁlms were digitally acquired with a BioDoc-200 It Imaging System (UVP, Waltham, MA), and densitometric analyses were performed with the LabWorks software (UVP).

### Open field test

An open field test was used pre-ischemia and at 24 hrs and five days after BCCAO for assessing exploratory and locomotor activity with an automated system (Electronic Motility Meter 40Fc; Motron Products, Sweden). Horizontal and vertical movements were recorded for a single 5-minute session. The total ambulatory distance was recorded as an index of locomotor activity, and the pattern of exploration was used as an index of reactions to stimulate situations that demand cognitive knowledge [30]. The open field arena was cleaned with 70% ethanol between trials.

### Experimental design and statistical tests

The sample size for each experiment is provided in the corresponding figure legend and represents the number of independent biological replicates (n), defined as individual mice. Data are presented as mean ± S.E.M. For comparisons of normally distributed data between two groups, an unpaired Student’s t-test was used. For comparisons across more than two groups, a one-way analysis of variance (ANOVA) was performed, followed by Tukey’s post-hoc test for multiple comparisons. Values of *p < 0.05, **p < 0.01, ***p < 0.001 were considered statistically significant. All statistical analyses were performed using GraphPad Prism (version 10.0.0).

## Results

### Effects of brain ischemia on the ultrastructure of the hippocampus CA1 after BCCAO, a comparison between AhR^+/+^ and AhR^-/-^ mice

We first evaluated the morphological changes in the nucleus (Nu) of neurons in the CA1 of AhR^+/+^ and AhR^-/-^ mice five days post-ischemia (Fig 1B). Both AhR^+/+^ and AhR^-/-^ control mice show that neuronal chromatin remained unchanged, and its material dispersed throughout the nucleus. However, in AhR^+/+^ mice, BCCAO caused peripheral chromatin condensation in the nucleus. In contrast, in BCCAO AhR^-/-^ mice, the nucleus showed a decrease of peripheral chromatin below the nuclear envelope and neuronal pyknosis (Fig 1C). Effects of brain ischemia revealed different structural patterns in endothelial cells near the CA1 region (Fig 1D). The control groups showed intact cytoplasm and mitochondria (Mit). Intact endothelial cells with continuous basement membranes (B.M.) and apparent tight junctions (red arrows) were observed (Fig 1B). We investigated whether the distention of endothelial cells was necessary for the formation of larger diameter capillaries, specifically if larger capillaries had thinner walls due to cell stretching. We found that larger capillaries actually had thicker endothelial walls. The AhR^+/+^ and AhR^-/-^ control groups did not show changes, however the ischemic AhR^+/+^ group had thinner walls compared with both AhR^+/+^ control (***p = 0.0002) and AhR^-/-^ control (*p = 0.0125). Interestingly, the AhR^-/-^ ischemic group showed an increase in endothelial thickness compared to the AhR^+/+^ Ischemic group (***p = 0.0004). Other characteristics observed were normal Astrocyte processes (A.P.) around the endothelium and the capillary lumen (L) (Fig 1B). The neurovascular unit of BCCAO AhR^+/+^ mice exhibited severe edema, altered BM and collapsed AP (asterisk). Whereas edema was reduced, and a continuous basement membrane was observed in BCCAO AhR^-/-^ mice. The capillary lumen and AP regular structures, and the mitochondria did not show significant alterations. Myelinating fibers and axons observed at the ultrastructural level in both AhR^+/+^ and AhR^-/-^ control mice appear normal with plasma membrane rings of regular sizes and densities (Fig 1E). Axonal degeneration was evident in the proportion of reduced myelin sheath in the BCCAO AhR^+/+^ group (*p = 0.0332) compared to AhR^+/+^ Control and (**p = 0.0022) compared to AhR^-/-^ control groups. In contrast, although demyelination was observed in the BCCAO AhR^-/-^, it was not as severe as in the AhR^+/+^ group. Interestingly, there was a decrease in demyelination in AhR^-/-^ mice five days after brain injury (Fig 1E). Furthermore, synapses were identified by a presynaptic density in the layer of CA1 with the presence of synaptic vesicles near the membrane. There were no changes in synapse density between control groups (Fig 1F). However, we observed a 68% lower density of synapses in BCCAO AhR^+/+^ and BCCAO AhR^-/-^ mice (Fig 1F) (***p = 0.0002). Interestingly, the synapses’ morphological features differed between AhR^+/+^ and AhR^-/-^ ischemic mice. The formation of the synaptic cleft and the postsynaptic density (arrows indicate synapse junctions) show higher postsynaptic density length (arrowhead) but

lower number of synapses in AhR^+/+^ CA1 compared to AhR^-/-^ CA1 (thin arrow). In addition, discontinuous postsynaptic density possibly reflects mature synapses that have undergone structural changes because of brain injury (indicated by arrows) (Fig 1B).

### Rsv-treatment or the absence of aryl hydrocarbon receptor protects against neurodegeneration and reduced astrogliosis after BCCAO

**Fig 2:**
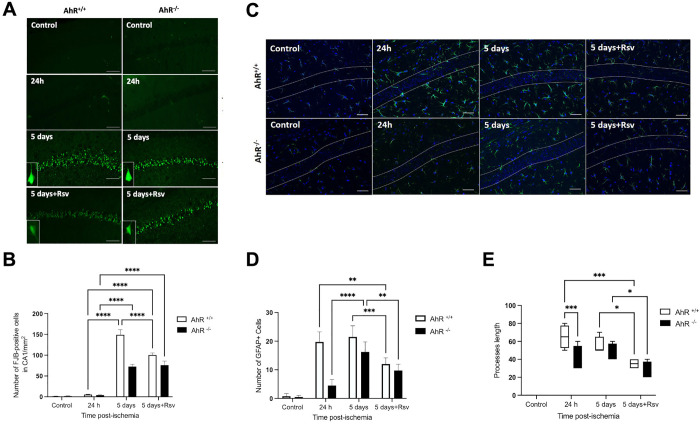
A) Fluoro-Jade B staining in hippocampal CA1 of AhR + /+ and AhR -/- mice after ischemic injury treated with Rsv. **B)** Quantitative analysis of FJB-positive cells in the hippocampal CA1 region five days post-ischemia. Data are presented as means ± **S.**E.M (n = 5 independent biological replicates per group). Statistical significance was determined by one-way ANOVA followed by Tukey’s multiple-comparison test. *p < 0.05, **p < 0.01 compared to the BCCAO AhR^+/+^ group. **C)** Astrogliosis alterations in the hippocampus CA1 after BCCAO. We assessed GFAP immunofluorescence in the CA1 in both AhR^+/+^ and AhR^-/-^ mice to determine astrogliosis. Ischemic injury in AhR^+/+^ mice causes a significant increase in astrogliosis, whereas a lower expression of GFAP is observed in both AhR^+/+^ and AhR^-/-^ mice treated with Rsv. n = 5 per group, *p < 0.0240; *p < 0.0172. Scale bars = 50µm. **D)** Quantitative analysis of GFAP-positive cells in the hippocampal CA1 region five days post-ischemia. *p < 0.05, **p < 0.01, ***p < 0.001, ****p < 0.0001 compared to the BCCAO AhR^+/+^ **E)**. For the Sholl analysis, quantitative analysis of processes length cells in the hippocampal CA1 region five days post-ischemia. *p < 0.05, **p < 0.01, ***p < 0.001, compared to the BCCAO AhR^+/+^.

We evaluated neurodegeneration by Fluoro-Jade B staining (Fig 2A). The control groups (AhR^*+/+*^ and AhR^-/-^) did not receive bilateral carotid occlusion and 24h after the procedure the ischemic groups did not present Fluoro-Jade B-positive cells. By contrast, 5 days after carotid occlusion, we observed neuronal degeneration in both AhR^*+/+*^ and AhR^-/-^ ischemic groups (****p < 0.0001). Statistically more Fluoro-Jade B-positive cells are present in AhR^*+/+*^ mice than in AhR^-/-^ mice and Rsv-treated groups (****p < 0.0001) Nevertheless, the AhR^-/-^ mice without Rsv showed even fewer degenerated neurons in comparison to the Rsv-treatment groups (****p < 0.0001) (Fig 2B). Astrogliosis is a phenomenon that occurs after ischemia. The GFAP-positive cells are represented (Fig 2C). There was no difference between AhR^+/+^ and AhR^-/-^ control mice in GFAP-staining, nor in those which had received Rsv (Fig 2D). However, AhR^*+/+*^ groups show a very intense GFAP-staining at both 24 h (***p = 0.0017) and 5 days (****p < 0.0001) after damage around the hippocampus CA1 region compared to AhR^-/-^ groups Furthermore, in Rsv-treated groups (AhR^*+/+*^ and AhR^-/-^ mice) there is less astrogliosis significative statistic than in the other groups (****p < 0.0001) (Fig 2D). For the Sholl analysis, we drew a series of concentric circles centered on the astrocyte cell body immunostained for GFAP (See supplementary methods in S1 File). We were able to evaluate the length and complexity of the astrocyte processes. Larger cellular processes were observed in the BCCAO AhR^+/+^ group than in the BCCAO AhR^-/-^ group (***p < 0.001). In the five days post-ischemia there were no differences in both AhR^+/+^ and AhR^-/-^ mice. While, compared with groups of Rsv-treated if there was a difference (*p < 0.05) (Fig 2E).

### The expression of HIF-1α in hippocampus is higher in the absence of AhR compared to AhR^+/+^ mice

**Fig 3:**
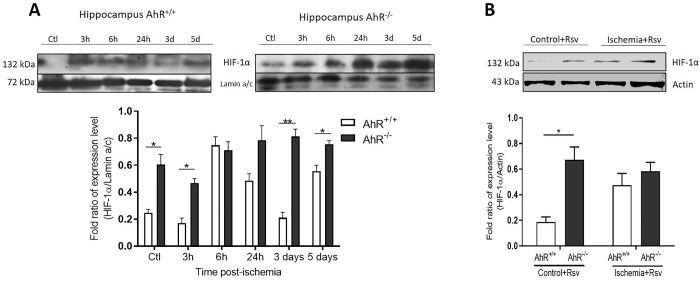
Expression of HIF-1 in the hippocampus. HIF-1α expression is modulated by AhR status. HIF-1α expression in CA1 hippocampal lysates from AhR^+/+^ and AhR^-/-^ mice at various times after BCCAO (purified nuclear fractions). The use of nuclear fractions confirms the specific nuclear localization of the protein. See Supplementary Figure S1 in S1 File for validation of fraction purity. **A)** Representative western blots of HIF-1a. Graph shows western blot analysis of HIF-1α expression 3, 6, 24 h, 3 and 5 days post-ischemic injury, showing a significant increase in protein-expression in the *AhR*^*-/-*^ group at different times. **B)** Western blot analysis of HIF-1α expression at 5 days post-injury in both AhR^*+/+*^ and *AhR*^*-/-*^ mice under Rsv treatment. n = 4-5 per group. *P < 0.05; **P < 0.01 (Student t-test).

Low oxygen levels lead to HIF-1α expression; therefore, the regulation of this protein is associated with neuroprotection. HIF-1α expression in AhR^+/+^ and AhR^-/-^ mice after the ischemic insult is shown in Fig 3A. The AhR^-/-^ control group had a significantly enhanced HIF-1α expression compared to the AhR^+/+^ control group as well as at the three-h post-ischemia group (*p = 0.0242). HIF-1α expression showed no difference between 6 and 24 h after BCCAO. However, three days post-damage, AhR^-/-^ mice showed a significant increase in HIF-1α expression compared to AhR^+/+^ mice (**p = 0.0028). Furthermore, increased HIF-1α protein expression is constant five days after ischemia (*p = 0.047). In contrast, we found differences in HIF-1α expression between AhR^+/+^ and AhR^-/-^ control groups followed by Rsv treatment (*p = 0.0188); however, five days post-ischemia there was no difference (Fig 3B).

### Neuronal and inducible nitric oxide synthases exhibit differential expression patterns in hippocampus of AhR^+/+^ and AhR^-/-^ mice after BCCAO and treated with Rsv

**Fig 4:**
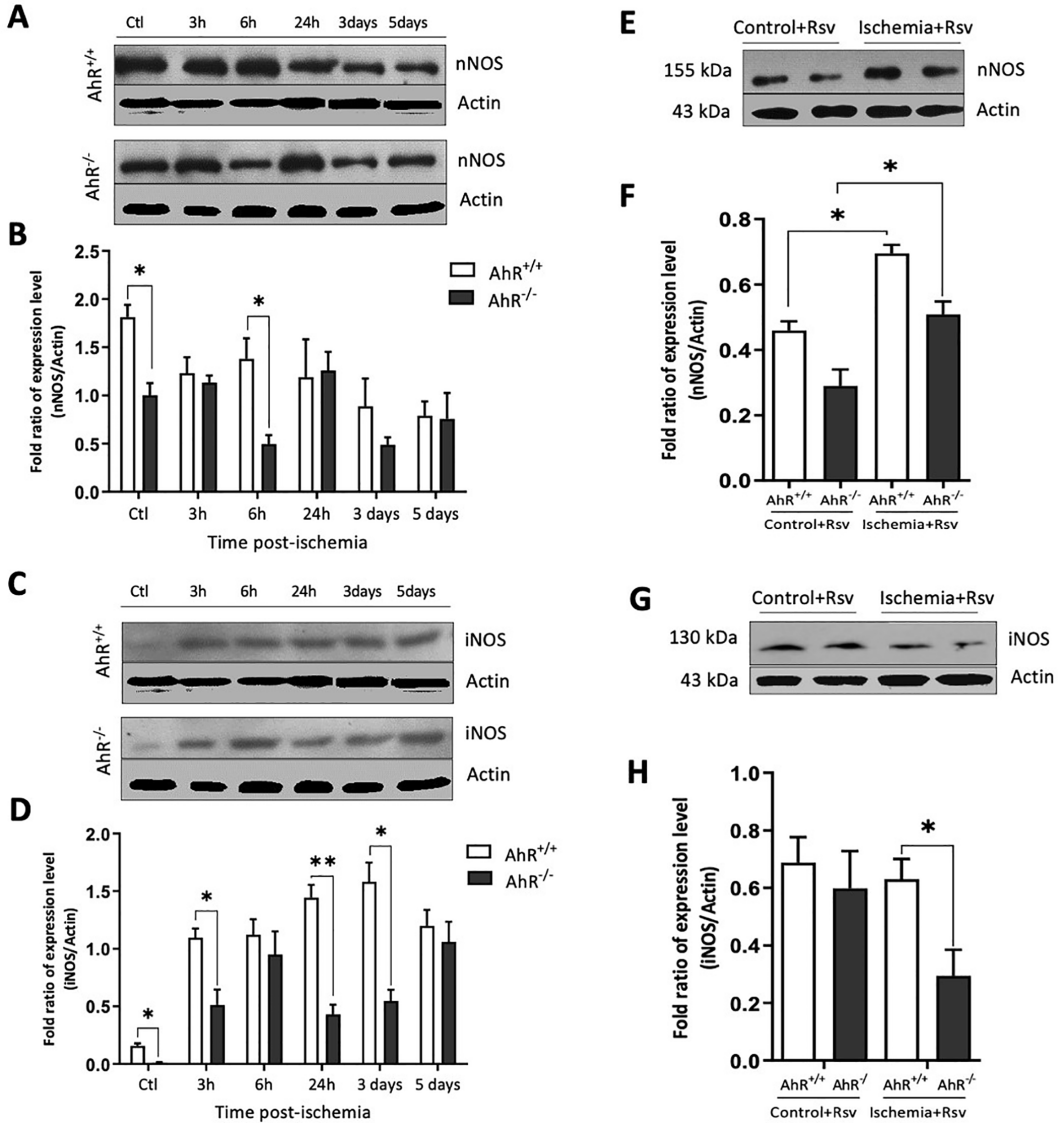
Ischemic damage by BCCAO induces different expressions of nNOS and iNOS in both AhR + /+ and AhR-/- mice. **A** and **B)** Representative western blot of nNOS and iNOS protein in the hippocampus at different times post-ischemia (3, 6, 24 h, 3 and 5 days) and graph shows the ratio of nNOS/Actin and iNOS/Actin in AhR + /+ and AhR-/- mice groups. **C)** Levels of nNOS expression increased in the hippocampus of AhR + / + mice treated with Rsv (control and ischemia), compared with the hippocampus of AhR–/– mice with Rsv (control and ischemia). **D)** Hippocampal expression of iNOS decreased in the Rsv treatment in AhR–/– mice compared to the hippocampus of AhR + / + mice as determined by Western Blot analysis. n = 4-5 per group. *p < 0.05; **p < 0.01 (Student t-test). Data are expressed as fold-change relative to baseline control.

We evaluated the expression of nNOS and iNOS in AhR^+/+^ and AhR^-/-^ mice after ischemia and treatment with Rsv (Fig 4). The expression of nNOS in the hippocampus of AhR^-/-^ mice was lower than in the AhR^+/+^ mice in control and six h post-ischemia (*p = 0.046). However, there were no significant changes between AhR^+/+^ and AhR^-/-^ mice (Fig 4B). In contrast, iNOS expression in AhR^-/-^ mice showed significant differences before and after BCCAO (3 h, 24 h, and three days), respectively (*p = 0.047; **p = 0.0089) compared with AhR^+/+^ mice. At six h and five days post-ischemia, there were no differences in iNOS expression in AhR^+/+^ vs AhR^-/-^ mice (Fig 4D). On the other hand, there was an increased expression of nNOS in the AhR^+/+^ ischemic group compared with the AhR^+/+^ control group treated with Rsv (*p < 0.0171), as well as in the hippocampus of AhR^-/-^ mice treated with Rsv (*p = 0.0111) (Fig 4F). Neither control nor ischemic groups that received Rsv treatment showed significant changes between AhR^+/+^ and AhR^-/-^ control groups in nNOS expression. Instead, Rsv inhibited the expression of nNOS induced by the BCCAO in the AhR^-/-^ group compared to the AhR^+/+^ group (*p = 0.0211) (Fig 4F). Whereas the expression of iNOS reduced significantly by Rsv induced by the BCCAO in the AhR^-/-^ group compared to the AhR^+/+^ group (*p < 0.05).

### BCCAO and Rsv modified KATII, claudin 5, and IL-6 expression in the hippocampus of AhR^+/+^ and AhR^-/-^ mice

**Fig 5:**
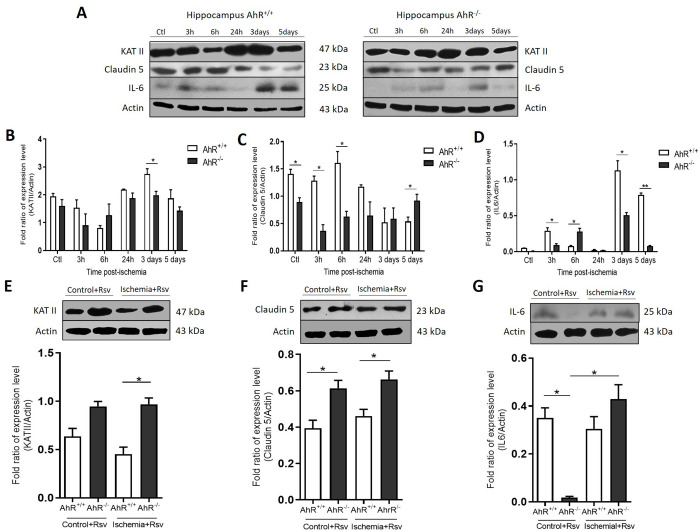
The hippocampus of AhR-/- mice showed differences in the expression patterns of KATII, Claudin 5, and IL-6 compared with AhR + / + mice after injury and treated with Rsv. **A)** Western blot of the proteins previously mentioned at different times (3, 6, 24h, 3 and 5 days) post-ischemia. **B)** Analysis of protein expression of KATII, claudin 5, and IL6 expression was determined in the hippocampus of AhR + /+ and AhR–/– mice. Rsv modifies the expression of KATII, claudin 5, and IL-6 after an ischemic lesion. **C)** No significant change in the control groups (AhR + /+ and AhR–/– mice), whereas the expression of KATII increased considerably in the ischemic group of AhR–/– mice. **D)** Significant changes of claudin 5 increased in both experimental groups (control and ischemia) of AhR–/– mice after Rsv administration. **E)** IL-6 expression in the AhR–/– control group after Rsv treatment; however, the expression of IL-6 increased after bilateral carotid occlusion in the AhR–/–group treated with the Rsv at five days. n = 4-5 per group. *p < 0.05; **p < 0.01 (Student t-test).

We determined the expression of KATII, claudin 5, and IL-6 (Fig 5A). KATII is responsible for the synthesis of KYNA, and we did not observe significant changes in KATII expression either before or immediately after ischemic injury; only three days after surgery, we found significant changes in the AhR^+/+^ ischemic group compared with AhR^-/-^ mice (*p = 0.0258) (Fig 5B). Furthermore, we decided to evaluate the expression of claudin 5, a protein implicated with BBB functioning. Interestingly, we observed in both the AhR^-/-^ control and the AhR^-/-^ ischemic groups an apparent reduction in the expression of claudin 5 compared to AhR^+/+^ groups six h after injury. However, five days post-ischemia, claudin 5 was higher in the hippocampus of AhR^-/-^ mice compared to AhR^+/+^ mice (*p = 0.0348) (Fig 5C). On another note, we evaluated the expression of key inflammatory cytokines. The expression of IL-6 was compared between AhR^+/+^ and AhR^-/-^ mice three h post-injury, and we observed a significant increase of IL-6 expression in AhR^+/+^ mice compared to AhR^-/-^ mice (*p = 0.0274). Whereas six h post-ischemia, an increased expression was observed in the hippocampus of AhR^-/-^ mice. While these fold-changes may appear modest, in the context of cerebral ischemia, sustained shifts in the levels of pivotal cytokines like IL-6 are known to significantly influence the inflammatory microenvironment, neuronal function, and recovery trajectories. Furthermore, we found that IL-6 expression was higher 3- and 5-day post-ischemia in the hippocampus of AhR^+/+^ mice compared with AhR^-/-^ mice (*p = 0.0377; **p = 0.0091) (Fig 5D), supporting the role of AhR in exacerbating the neuroinflammatory response to ischemia.

To assess whether the administration of Rsv affected KATII, claudin 5, and IL-6, we evaluated their expression in the hippocampus and compared AhR^+/+^ mice with AhR^-/-^ mice. In control groups, KATII increased; however, it was not significant compared to AhR^-/-^ mice. Interestingly, in the AhR^-/-^ ischemic group, KATII expression was higher five days after ischemia and Rsv treatment in comparison with the AhR^+/+^ ischemic group (*p = 0.0155) (Fig 5E). The expression of claudin 5 was higher in both groups of AhR^-/-^ mice treated with Rsv compared to AhR^+/+^ groups; in the hippocampus of the AhR^-/-^ control group, a significantly decreased expression of 80% in AhR^-/-^ control group was determined in comparison with pre-treatment to the pre-treatment with Rsv (*p = 0.0380) (Fig 5F). Conversely, after the ischemic event and having received Rsv, there was a significant increase in IL-6 expression compared with its homologous control (*p = 0.0254) but not in the AhR^+/+^ ischemic group (Fig 5G).

### Exploratory and locomotor activity improved in both AhR^+/+^ and AhR^-/-^ mice treated with Rsv after ischemic injury

**Fig 6:**
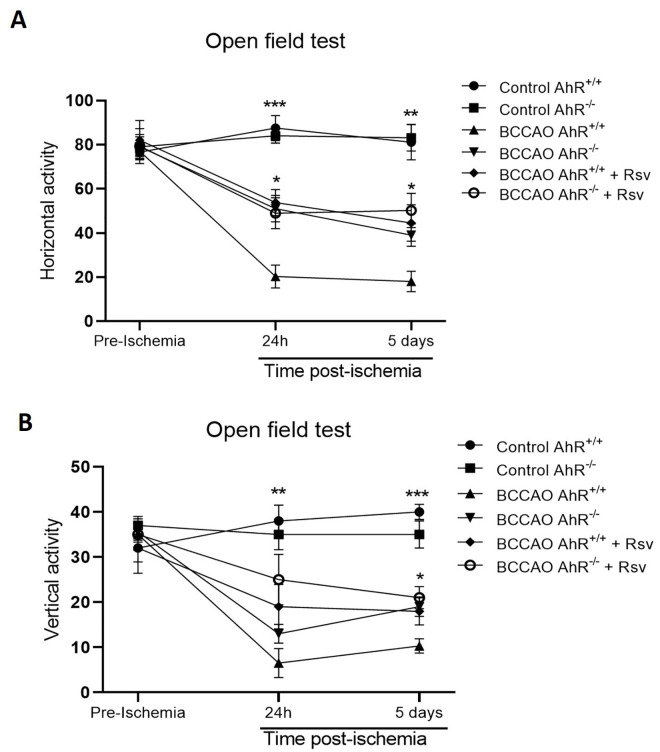
The activity of mice in the open field test after BCCAO. **A)** Shows the horizontal locomotor activity and **(B)** the vertical activity of mice in the open field test for 5 min. Time spent in the open field did not differ between AhR + /+ and AhR-/- mice before injury (Pre-ischemia). However, after ischemic damage (24 h and five days) (BCCAO AhR + /+), we observed a decrease in locomotor and exploratory activities in AhR + / + mice compared with Control groups (AhR + /+ and AhR-/-). In contrast, AhR + /+ and AhR-/- mice treated with the Rsv (BCCAO AhR + /+ + Rsv and BCCAO AhR-/- + Rsv) improved their motor activity. Data are mean ± SEM and were analyzed using one-way ANOVA and Bonferroni post-hoc test; n = 4-5 mice per group *P < 0.05; **P < 0.01; ***P < 0.001.

We evaluated motor activity using an open field test 24 h and five days after brain ischemia and of mice with and without Rsv (Fig 6). Data shows that the ischemic event decreases the exploration and locomotor activities compared to the control groups (**p = 0.0049, ***p = 0.00093). In contrast, both the pharmacological blockade with Rsv in AhR^+/+^ and AhR^-/-^ after ischemia improved locomotor activity 24 h and five days later compared with the ischemic-AhR^+/+^ group (*p = 0.0149) (Fig 6A). Nevertheless, there was no difference between AhR^+/+^ plus Rsv vs AhR^-/-^ without Rsv (Fig 6B). On another note, AhR^-/-^ mice did not show any difference in motor activity between treated and non-treated groups at any time.

## Discussion

The AhR plays an essential role in physiological and pathological phenomena by modulating signaling pathways [1]. Resveratrol has been described as an AhR antagonist, yet it is a pleiotropic compound with multiple AhR-independent actions, that can produce anti-inflammatory or metabolic effects overlapping with AhR blockade [31,32]. The results presented here support the hypothesis that the absence or pharmacological blockade of the AhR promotes a neuroprotective effect, and the data allows us to suggest the potential of the pharmacological modulation of the activity of the AhR in ischemia treatment [33].

The first part of this work showed severe nuclear alterations in neurons of the CA1 of AhR^+/+^ mice after brain ischemia and, to a lesser extent, in AhR^-/-^ ischemic mice. Ischemic stroke implies the obstruction of blood vessels; thus, blood flow must be restored to regain normal brain functioning [34]. However, BCCAO-induced ischemia leads to severe microvessel injury in AhR^+/+^ mice. The physiological and pathophysiological functioning of the hippocampus is shaped by its vasculature, and it is very sensitive to hypoxia [35]. Interestingly, in AhR^-/-^ mice, the fine hippocampal capillaries are less affected.

After stroke, damage to the myelin sheath is closely related to the vulnerability of nerve connections [34]. We found demyelination in the AhR^+/+^ ischemic group, consistent with previous studies. However, in the AhR^-/-^ ischemic mice, few disrupted myelin sheaths with stratification, collapse, and disruption were observed. In this regard, it has been reported that the excitotoxic process activated during ischemia leads to myelin injury and that a knockdown or blocking of the aryl hydrocarbon receptor mitigates excitotoxicity [36], and this could be related to less damage to myelin.

The pathogenic events of neurodegenerative diseases are triggered by reductions in the number of synapses [37]. Brain ischemia significantly reduced the number of synapses in the dendritic layer of CA1 both in AhR^+/+^ and AhR^-/-^ ischemic groups; interestingly, a significant difference emerged between control and ischemic animals. Furthermore, we found that synaptic membranes increased in size and number of parameters in the AhR^-/-^ mice, probably to compensate for the loss of synapses. Synaptic contacts are critical functional elements of the hippocampus, and the synaptic remodeling includes changes in the width of the synaptic cleft, length of the active zones, and postsynaptic density thickness [38]. We found these same findings in the AhR^-/-^ ischemic group (Fig 1F).

Thus, we examined whether neurodegeneration was prevented in the AhR^-/-^ mice. We found degenerated neurons mainly in the AhR^+/+^ ischemic group and observed that the Rsv-treatment prevented the loss of the pyramidal cells in the CA1 layer, a finding consistent with the report of Simao et al. [39] (Fig 2A). In other brain areas, Rsv also promotes neuroprotective following a nigrostriatal pathway injury [40]. However, an increase in the protection of AhR^-/-^ treated with Rsv was not observed, indicating that the AhR^-/-^, *per se*, facilitates a neuroprotective effect.

The activation of the AhR induces inflammation and gliosis. In our study, the CA1 hippocampal layer of the AhR^+/+^ ischemic mice showed a very intense GFAP staining 24 h and five days after the insult, indicating severe damage, in contrast to the AhR^-/-^ ischemic group that showed a lower GFAP response. A pertinent question arising from our study relates to the cell-type-specific role of AhR in mediating hippocampal injury. Previous studies have reported that AhR nuclear translocation differs among neurons, astrocytes, and microglia, driving distinct transcriptional programs that influence both inflammatory responses and neuronal injury in models of cerebral ischemia [41,42]. For instance, in astrocytes, AhR activation has been linked to enhanced gliosis and reduced neurogenesis [43], whereas in microglia, it regulates the production of pro-inflammatory cytokines [44]. Although we did not evaluate cell-type–specific AhR nuclear localization in the present study, future experiments using immunofluorescence with cell-specific markers or nuclear/cytosolic fractionation will be essential to determine which cell populations mediate the observed effects.

Signaling induced by cerebral ischemia may activate astrogliosis, contributing to tissue damage and neuroinflammation [45]. The AhR-mediated pathway participates in the pro-migratory effects of astrocyte activation. However, the Rsv-treatment in both AhR^+/+^ and AhR^-/-^ ischemic groups similarly reduces the number of GFAP-positive cell. Cerebral ischemia-induced signaling activates astrogliosis, which contributes to tissue damage and neuroinflammation [45]. While the AhR pathway has been implicated in the pro-migratory effects of astrocyte activation, our data suggest that resveratrol (Rsv) attenuates ischemia-induced astrogliosis through an AhR-independent mechanism. This is evidenced by our finding that Rsv treatment similarly reduced the number of GFAP-positive cells in both AhR + /+ and AhR-/- ischemic mice. This effect is consistent with previous reports demonstrating Rsv’s capacity to attenuate gliosis, for example, in retinal ganglion damage by reducing microglial activation [46].

Previous reports showed the Rsv capacity to attenuate gliosis in retinal ganglion damage due to its ability to reduce microglial activation [46].

Moreover, HIF-1α promotes the expression of genes associated with cell survival under hypoxia and ischemia [47]. We found elevated levels of HIF1α in the hippocampus of AhR^-/-^ mice compared with AhR^+/+^ mice. Furthermore, both HIF-1α and AhR are sensors of environmental cues and share the obligatory heterodimer partner ARNT. The expression levels and availability of ARNT can modulate the transcriptional output of both pathways [48]. Moreover, competition and crosstalk between the AhR and HIF pathways have been reported, especially in ischemic models where hypoxia and HIF-1α stabilization could change ARNT dynamics [49]. This potential interaction is a critical consideration for interpreting AhR activity in our ischemia model, as the transcriptional consequences of AhR activation or inhibition may be influenced by this crosstalk [50]. Interestingly, the results of our study add new evidence indicating that Rsv does not affect HIF-1α after ischemic injury in either the hippocampus of AhR^+/+^ or AhR^-/-^ mice. This could be due to the various biological effects of Rsv. One limitation of this study is that the Resveratrol treatment was not evaluated at the 3-day post-ischemia time point, where the impact of AhR^-/-^ on HIF-1α was most pronounced. Future investigations should include this earlier time point to fully characterize the temporal dynamics of pharmacological AhR blockade. The present data indicated that Rsv prevents damage independently of HIF-1α.

It is well known that nitric oxide has both protective and deleterious effects during ischemia, depending on the type of NOS isoform secreted by neurons [51,52]. In this study, the elevation of hippocampal nNOS levels found in AhR^+/+^ mice compared with the AhR^-/-^ mice after brain ischemia agrees with a previous report [53]. The expression of nNOS is lower in AhR^-/-^ mice; this could be associated with the regulation by AhR. On the other hand, the early expression of iNOS is classically associated with the damage. The BCCAO augments the expression levels of iNOS at 3, 24 h, and three days after injury in the hippocampus of AhR^+/+^ animals. Nevertheless, it is not higher in AhR^-/-^ mice. Generally, the regulation of the expression of iNOS occurs through the activation of STAT1. Previous evidence from inflammatory phenomena suggests the formation of a complex between AhR and Stat 1 [54]. Lack of AhR or the use of Rsv attenuates the expression of iNOS after ischemic injury. Rsv possesses antioxidant effects and is considered an agent with neuroprotective potential for treating focal cerebral ischemia injury [55].

The altered levels of KYNA are implicated in the pathophysiology of several neurodegenerative diseases [56,57]. Thus, we determined the expression levels of KATII, the de novo KYNA synthesis enzyme, in the hippocampus of both control and knock-out mice. Three days post-injury we observed a tendency of a higher expression of the enzyme in the hippocampus of AhR-/- mice compared to AhR^+/+^ mice. Interestingly, it also appears to be a decrease of the enzyme in the Rsv-treated wild-type mice. Thus, the absence of the AhR may increase the availability of KYNA, consequently promoting beneficial effects; however, further work is necessary to demonstrate this hypothesis.

Under ischemic conditions, the impairment of the BBB integrity is associated with molecular alterations of tight junctions [58]. In the present study, five days after ischemic damage, the expression of claudin 5 significantly increased in AhR^-/-^ mice and decreased in AhR^+/+^ mice. The activities of COX have a critical role in tight junction dysregulation [59], and the AhR affects target genes such as COX2; thus, the absence of the AhR could explain the 5-day-post-ischemia increase in claudin 5 associated with BBB recovery. Interestingly, AhR^-/-^ control mice and mice treated with Rsv showed an increase of claudin 5 when compared with AhR^+/+^ mice. Several studies reported that Rsv restores neuronal tight junctions, including claudin 5, which improves the integrity of the disrupted BBB [59].

Inflammation is critical in the pathophysiology of ischemic stroke. While the observed fold-changes in IL-6, in our model may be modest, their biological significance is substantial [60]. The brain’s inflammatory milieu is a tightly regulated system, and even small, sustained alterations in key mediators like IL-6, TNF-α, and IL-1β can tip the balance between neuroprotection and neurodegeneration, influencing glial activation, blood-brain barrier integrity, and ultimately, functional outcome [61]. In our study, the expression of IL-6 was generally heightened in AhR^+/+^ mice after ischemia, a response that was attenuated in AhR^-/-^ mice (Fig 5). This pattern is consistent with the established role of AhR as a modulator of immune and inflammatory responses. The AhR regulates inflammatory cytokines in many diseases driven by immune/inflammatory processes, and its absence appears to foster a less hostile inflammatory environment post-ischemia [62]. The AhR is widely recognized as a potent regulator of inflammation, and its deficiency can lead to a dysregulated inflammatory response; however, in the acute phase of cerebral ischemia, our data suggests its absence mitigates the production of key pro-inflammatory cytokines. A subtle change in AhR activation can lead to a substantial increase in the transcription of its target genes, such as CYP1A1, CYP1B1, and various inflammatory mediators. This explains how a minor shift in upstream signaling can produce a significant downstream phenotypic effect [63]. The effects of the long-term regulation of the AhR on different signaling pathways need to be further explored. The neuroprotective effect of Rsv, which reduces damage, could occur because it promotes the expression of IL6, inducing the classic pathway.

In several neurodegenerative diseases, motor function is assessed by performing behavioral tests [64]. In the present study, AhR^+/+^ and AhR^-/-^ mice receiving Rsv after ischemic injury displayed significantly higher motor activity than those in the AhR^+/+^ ischemic group at 24 h and five days. This suggests a significant favorable recovery. Some reports have shown the importance of the test to assess skilled walking impairments after ischemic and hemorrhagic to identify the effects of different rehabilitation protocols [65,66]. The reasons for these behavioral differences need to be better understood and may be mainly related to the lack of the AhR combined with the Rsv. A previous study showed that AhR^-/-^ mice remained on the rotarod (motor coordination test) for a longer time compared to AhR^+/+^ mice [6]. The constitutive activation of the AhR signaling drastically disrupts the dendritic arborization of pyramidal neurons during hippocampal development [5]; this could be related to motor ability. While resveratrol inhibits canonical AhR target gene induction under our conditions, it is crucial to acknowledge its limitations as a specific pharmacological tool. Resveratrol exerts several AhR-independent effects, which may confound the interpretation of its role as an AhR antagonist [67–69]. Its pleiotropic nature means that the neuroprotection observed in our study likely reflects a combination of AhR-dependent and AhR-independent mechanisms. For instance, the protective effects seen in AhR^-/-^ mice treated with Rsv are consistent with its known antioxidant and anti-inflammatory properties acting through other pathways [67,70]. Therefore, although our data is consistent with AhR involvement, we cannot exclude contributions from AhR-independent mechanisms. Future studies employing more selective AhR antagonists, such as CH223191, which has been widely used as a pharmacological tool to confirm AhR-specific effects, are required to establish causality and better dissect the AHR-specific component of the neuroprotective phenotype.

## Conclusion

The most remarkable finding of this study is that the absence or the blockade of the AhR reduced the progression of the damage caused by brain ischemia since the absence of the AhR prevents the activation of harmful signaling in the hippocampus. Therefore, the expression of HIF-1α might have a role in viable neurons to upregulate another neuroprotective mechanism. Additionally, we demonstrated a novel aspect of the pleiotropic actions of Rsv since it acts as an AhR-inhibitor promoting neuroprotection that improves the integrity and functioning of the Blood-Brain Barrier and reduces oxidative and inflammatory activities without implicating the canonical pathways.

## Supporting information

S1 File S1 Supporting Information regarding Electron microscopy is available. (DOCX)

## Abbreviations

AhR: Aryl hydrocarbon receptor
AhR^-/-^: AhR-null mice
AhR^+/+^: Homozygous wild-type mice
BBB: Blood-Brain Barrier
bHLH/PAS: basic helix loop helix-Per-Arnt-Sim
BCCAO: Bilateral Common Carotid Artery Occlusion
GFAP: Glial Fibrillary Acidic Protein
HIF-1: Hypoxia-inducible factor 1
iNOS: inducible Nitric Oxide Synthetase
IL-6: Interleukin 6
KAT II: Kynurenine aminotransferase II
nNOS: neuronal Nitric Oxide Synthetase
Rsv: Resveratrol
XME: xenobiotic-metabolizing enzyme
