# Supplementary material for: The absence or the pharmacological blockade of the aryl hydrocarbon receptor promotes neuroprotection in the hippocampus after an ischemic insult
Source: PLoS One. 2025 Dec 26;20(12):e0338936. doi: 10.1371/journal.pone.0338936 (PMC12742769; doi:10.1371/journal.pone.0338936)
Supplement: S1 File — (DOCX) [file pone.0338936.s001.docx]

Supplementary methods

1. **Quantitative Analysis of Chromatin Condensation by Electron Microscopy**. Chromatin condensation was quantified using transmission electron microscopy. In brief, samples were prepared by fixing cells in 2.5% glutaraldehyde, post-fixing them in 1% osmium tetroxide, and embedding them in epoxy resin. They were then stained with uranyl acetate and lead citrate, and images were captured at 10,000x magnification (Fig 1A). Image analysis was conducted using ImageJ software. The nuclear boundary was manually delineated to define the region of interest (ROI). Each micrograph was converted into an 8-bit grayscale image. To objectively segment condensed chromatin, a consistent thresholding algorithm (Otsu's method) was applied to the entire image set (Fig 1B). This method automatically separates the pixel histogram into dark, electron-dense heterochromatin and light, electron-lucent, euchromatin populations. The 'Analyze Particles' function was then utilized to calculate the percentage of the nuclear ROI area occupied by thresholder pixels, representing the heterochromatin area fraction. This value serves as a direct quantitative index of global nuclear chromatin compaction.


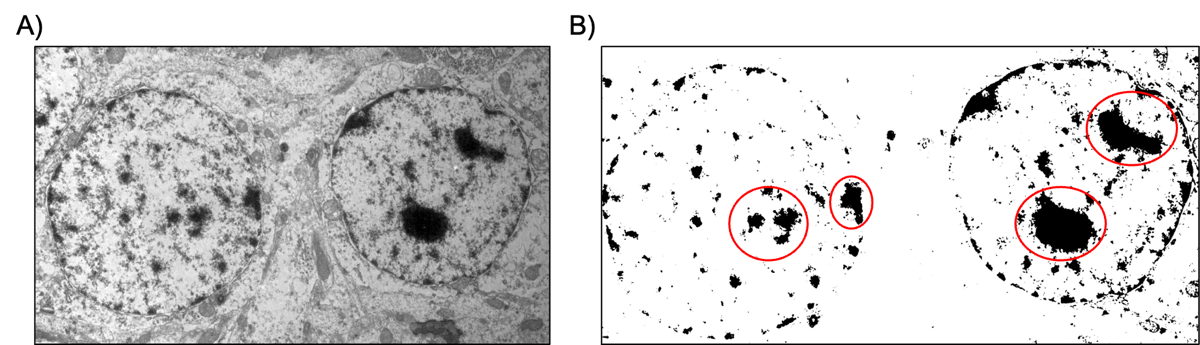


Figure 1. Chromatin condensation by TEM. A) Representative nuclei image captured by TEM, showing the electron-dense heterochromatin and electron-lucent, euchromatin in the hippocampus CA1 region. B) Micrograph converted into an 8-bit grayscale image and analyzing the pixel histogram. The red circle demarcates the nuclear boundary used for quantitative chromatin analysis. Pixel intensity within this region was thresholded to classify and quantify electron-dense areas as heterochromatin and electron-lucent areas as euchromatin.

1. **Myelin Decompaction Analysis:** We categorized the axons as normally myelinated, which included compact, electron-dense myelin sheaths. However, the decompacted myelin showed more than 15% of the myelin sheath with split lamellae and distinct layers. We measured the thickness of the myelin from the inner to the outer membrane. Subsequently, the axon diameters were divided by the total fiber diameter (axon + myelin) to assess myelin integrity relative to axon size, referred to as the g-ratio. We report the results using the MyelTracer Software, an installable, stand-alone software for semi-automated g-ratio quantification based on the Open Computer Vision Library (OpenCV) (<https://github.com/HarrisonAllen/MyelTracer>) that was published by Kaiser et al., 2021
2. B)


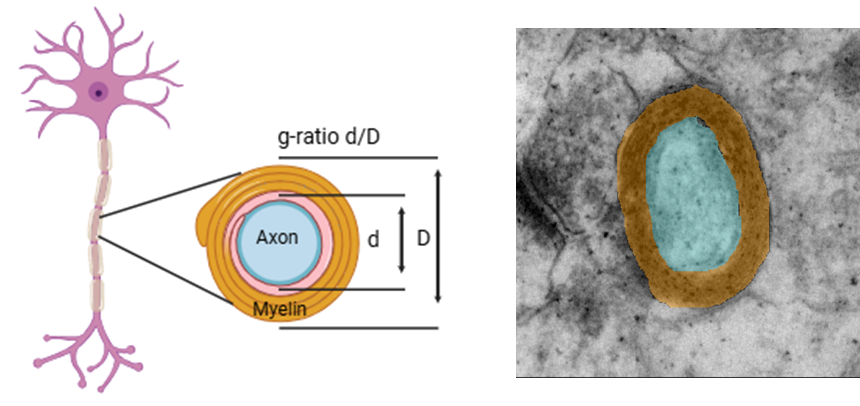


Figure 2. Quantitative analysis of myelination of TEM samples. A) Representative scheme of a myelinated axon and diagram and calculation of the g-ratio of myelinated axons. Diagram and calculation of the g-ratio of elongated myelinated axons. Data are represented as mean ± s.e.m. *p < 0.05. (I) Percentage distribution of myelinated axon size. (J) Scatter plot of individual g-ratio versus axons. Axons with a diameter > 0.3 mm were included for analysis.

1. **Endothelial Thickness quantification.** Micrographs of capillaries from the hippocampal CA1 region were collected using transmission electron microscopy at a standard magnification of 8,000x (n=4-5 mice per group, with 5-10 capillaries analyzed per mouse). On each micrograph, the following measurements were made using ImageJ software: Basal lamina thickness was measured in three equidistant locations around the capillary circumference, and an average was taken. <https://journals.sagepub.com/doi/10.1177/0271678X16629976>

**Western blot analysis was performed to validate the efficacy of our subcellular fractionation protocol.** The fractionation procedure was carried out using a differential centrifugation approach. Briefly, cells were homogenized in a hypotonic buffer (10 mM HEPES pH 7.9, 10 mM KCl, 1.5 mM MgCl2, 0.5 mM DTT) supplemented with protease and phosphatase inhibitors. The homogenate was centrifuged at low speed (800 x g for 10 min at 4°C) to pellet the nuclear fraction. The resulting supernatant was further centrifuged at high speed (16,000 x g for 20 min at 4°C) to obtain the cytoplasmic fraction (supernatant) and the organellar/membrane fraction (pellet). The initial nuclear pellet was purified by washing and centrifugation through a dense sucrose cushion. To confirm the purity of the isolated fractions, we probed for the nuclear envelope marker Lamin A/C and the cytoplasmic marker β-Actin. The immunoblot results demonstrated a clean separation, with Lamin A/C detected exclusively in the nuclear fraction and β-Actin found only in the cytoplasmic fraction. The absence of significant cross-contamination validates the integrity of our fractionation method and ensures the reliability of subsequent compartment-specific protein analyses.


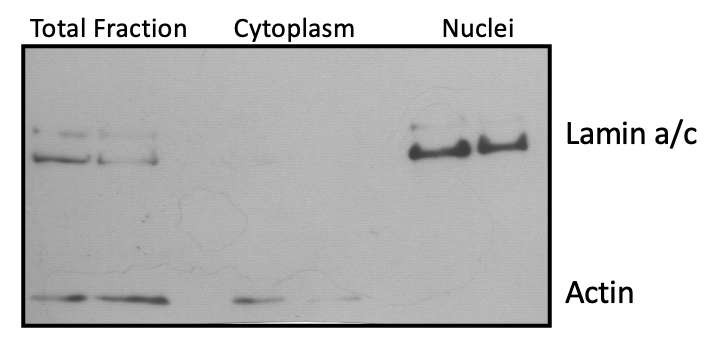


**Figure 4. Assessment of subcellular fractionation purity.** Representative western blots of proteins from cytoplasmic and nuclear fractions. The blot was probed for the nuclear envelope marker Lamin A/C and the cytoplasmic marker β-Actin. The precise compartmentalization of Lamin A/C to the nucleus and β-Actin to the cytoplasm validates the efficacy of the fractionation protocol. The lack of detectable Lamin A/C in the cytoplasmic fraction and the absence of β-Actin in the nuclear fraction confirm the successful isolation of highly enriched fractions and the absence of significant cross-contamination, ensuring the reliability of subsequent compartment-specific protein analyses.
